# Perceptions of, attitudes towards and barriers to male involvement in newborn care in rural Ghana, West Africa: a qualitative analysis

**DOI:** 10.1186/1471-2393-14-269

**Published:** 2014-08-12

**Authors:** Mari Dumbaugh, Charlotte Tawiah-Agyemang, Alexander Manu, Guus HA ten Asbroek, Betty Kirkwood, Zelee Hill

**Affiliations:** Institute for Global Health, University College London, 30 Guilford Street, London, WC1N 1EH UK; Kintampo Health Research Centre, Ghana Health Service, PO Box 200, Kintampo, Brong Ahafo Region Ghana; Department of Nutrition and Public Health Intervention Research, London School of Hygiene and Tropical Medicine, Keppel Street, London, WC1E 7HT UK

**Keywords:** Male involvement, Newborn care, Newborn health, Gender, Mother-in-law, Household roles, Birth preparedness, Care seeking, Ghana

## Abstract

**Background:**

Male involvement in various health practices is recognized as an important factor in improving maternal and child health outcomes. Male involvement interventions involve men in a variety of ways, at varying levels of inclusion and use a range of outcome measures. There is little agreement on how male involvement should be measured and some authors contend that male involvement may actually be detrimental to women’s empowerment and autonomy. Few studies explore the realities, perceptions, determinants and efficacy of male involvement in newborn care, especially in African contexts.

**Methods:**

Birth narratives of recent mothers (n = 25), in-depth interviews with recent fathers (n = 12) and two focus group discussions with fathers (n = 22) were conducted during the formative research phase of a community-based newborn care trial. Secondary analysis of this qualitative data identified emergent themes and established overall associations related to male involvement, newborn care and household roles in a rural African setting.

**Results:**

Data revealed that gender dictates many of the perceptions and politics surrounding newborn care in this context. The influence of mother-in-laws and generational power dynamics were also identified as significant. Women alone perform almost all tasks related to newborn care whereas men take on the traditional responsibilities of economic providers and decision makers, especially concerning their wives’ and children’s health. Most men were interested in being more involved in newborn care but identified barriers to increased involvement, many of which related to gendered and generational divisions of labour and space.

**Conclusions:**

Men defined involvement in a variety of ways, even if they were not physically involved in carrying out newborn care tasks. Some participant comments revealed potential risks of increasing male involvement suggesting that male involvement alone should not be an outcome in future interventions. Rather, the effect of male involvement on women’s autonomy, the dynamics of senior women’s influence and power and the real impact on health outcomes should be considered in intervention design and implementation. Any male involvement intervention should integrate a detailed understanding of context and strategies to include men in maternal and child health should be mutually empowering for both women and men.

## Background

Despite great gains in maternal and child health, 3.3 million newborns continue to die each year with 99% of deaths occurring in middle- and low-income countries [1]. Various interventions utilize male involvement to improve maternal and child health outcomes [2–15] however male involvement in newborn care is a relatively unexplored strategy.

A search of relevant databases for exploratory and intervention studies of male involvement in maternal and newborn health in African contexts published from 2000–2012 was completed. Search results revealed that male involvement interventions involve men in a variety of ways, at different levels of inclusion and use a range of outcome measures.

Reported outcomes of effective strategies to involve men in maternal and newborn health varied and included male attendance at maternal health services, men’s knowledge and attitudes, couple communication and relations, and service utilization of men and women such as treatment for sexually transmitted infections and HIV testing [3, 5, 9–14]. Partner notification is a widely-used strategy at the health facility level with significant results in a number of settings [3, 5, 9–11]. Interventions reported positive health behavior change in both men and women including increases in sexual and reproductive health service utilization, condom use, practices to prevent mother to child transmission of HIV, couple communication and reduced loss to follow up [3, 5, 9–11]. Although successful, partner notification can present challenges to increasing male involvement. Men are often reluctant to participate in clinic-based programmes as clinics are seen as women’s spaces; better understandings of barriers to male involvement and new tactics to include men in clinic settings are necessary [10].

Incentives were identified as an effective way to increase male involvement in antenatal care however this strategy was not sustainable and had some negative consequences [3]. For example, a UNICEF-sponsored competition in Malawi offered village chiefs a prize for high male attendance at antenatal care, but village chiefs subsequently discouraged health providers from seeing women who arrived to appointments without their husbands in order to win the prize [3]. An increase in male attendance at antenatal care was achieved at the expense of some women’s health.

Programmes addressing couple communication, health education and gender norms which incorporated men into intervention strategies from the beginning, sometimes placing men in leadership roles, were also successful [3, 12–14]. In contrast to traditional sexual and reproductive health education programmes which only distribute health information to women by women, these male-inclusive programmes encourage men and women to view each other as equal partners in health and relationships. Positive outcomes were reported in men’s sexual and reproductive health knowledge, investment and involvement in antenatal care and attitude changes around gender norms [3, 12–14].

Several exploratory and feasibility studies have investigated the potential for male involvement in HIV testing and prevention [7, 15, 16], sexual and reproductive health [17, 18] and safe motherhood initiatives [18–24]. The only exploratory study identified related to newborn care targeted preterm care [25].

Common barriers to male involvement identified across studies included lack of men’s maternal health knowledge, stigma of men who participate in maternal health services or family planning, and gendered divisions of cultural responsibilities, health structures, services and information [7, 15–24]. Most suggested strategies for overcoming barriers focused on education, empowerment and mobilization of women, men and the community as well as changing health structures and services to be more inclusive and enabling for men [7, 15–24].

Identified studies suggest that male involvement could play an important role in maternal and newborn health however evidence is limited and additional research is necessary. Using qualitative data collected from recent mothers and fathers in central rural Ghana, this paper seeks to fill a gap in the existing literature and inform future interventions by increasing knowledge and understanding of the potential for male involvement in newborn care in a rural African setting.

## Methods

Data were collected from December 2006-January 2007 in the Brong Ahafo Region, Ghana by trained, local fieldworkers during the formative research phase of a community-based newborn care trial, *NEWHINTS* [26]. *NEWHINTS* was a cluster randomized controlled trial aiming to affect behaviour change through home visits in pregnancy and the first week after birth as part of an integrated package [26]. *NEWHINTS* formative research methods [26], trial protocol including ethical approval [27] and outcomes [28] are available elsewhere. The original trial is registered with clinicaltrials.gov (identifier NCT00623337). Because this secondary analysis does not use data for purposes outside of objectives stated in the original protocol, University College London ethics committee did not require additional ethical approval.

For this paper we analyzed qualitative data sources from NEWHINTS formative research: 25 birth narratives of mothers who delivered at home in the two months previous to data collection and 12 in-depth interviews and 2 focus group discussions (FGD) with fathers. After giving written informed consent for participation, women were asked to share a detailed narrative of the most recent birth and describe practices and beliefs surrounding newborn care including the role of birth assistants and family members. The interviews and FGDs with consenting fathers included questions on fathers’ knowledge of newborn care practices, involvement in newborn care tasks and attitudes toward increasing their involvement in newborn care. Specific birth and newborn care practices that were explored with all participants included selecting delivery location, birth preparedness and complication readiness, drying and wrapping the baby after delivery, bathing, immediate and exclusive breastfeeding, cord care, care seeking and care for small babies.

Table 1 describes participant demographics.

**Table 1:** **Participant demographics**

Participant demographics			
	Women	Men	Men
		Interviews	Focus group discussions
# of participants	*n* = 25	*n* = 12	*n* = 22
Age range (years)	15-40*	20-50	30-80*
# of children	1-6	1-8	1-17+
Level of education			
None	*n* = 14	*n* = 3	*n* = 10
Primary	*n* = 1	*n* = 4	*n* = 5
Junior secondary	*n* = 8	*n* = 2	*n* = 5
Senior secondary	*n* = 1	*n* = 1	*n* = 2
Polytechnic or University	*n* = 0	*n* = 2	*n* = 0
Unknown	*n* = 1	*n* = 0	*n* = 0

*15 year old female and 80 year old male participants were outliers; representative ranges started at 18 years old for females and ended at 70 years old for males in focus groups. +17 children was an outlier; representative range for focus group discussion participants was between 1 and 9 children.

Participants were chosen from fourteen communities selected from three of six geo-political districts involved in the *NEWHINTS* trial; communities were selected to reflect study area diversity. Consenting mothers enrolled in a surveillance system were recruited for interviews. Fathers were recruited by word of mouth through local researchers. Participants were selected purposively to ensure a range of socio-demographic characteristics including age, ethnicity, education, parity and occupation. Fathers participating in FGDs tended to be older than those who were interviewed, however data analysis does not reveal associations between age and perceptions of male involvement or gender roles within the household. Interviews continued until saturation was reached. Data were collected in local languages through detailed field notes taken during interviews and FGDs . Fieldworker notes were transcribed into English on the same day as the interview or discussion. A sub-sample of interviews was tape recorded and checked for quality assurance.

Data coding and analysis was conducted manually by MD in collaboration with ZH. Reflexivity during coding and analysis was maintained through detailed researcher notes and frequent discussions with ZH who was responsible for supervising the formative research. All data was read several times for familiarisation. Specific themes correlating with study objectives were identified for investigation before analysis while additional themes and recurring associations emerged throughout the analytical process. Each theme/sub-theme was assigned a color code, data was manually sorted, and theoretical associations to gender dynamics, masculinities and household roles were established. All research and subsequent analysis meet the guidelines for Qualitative Research Review (RATS) as outlined by this journal (*see* biomedcentral.com).

## Results

Themes emerging from analysis related to current socio-cultural perceptions of male involvement as well as the potential for and possible challenges of increasing male involvement. Participant responses reflected strict gender-based divisions of work and space that relegated certain knowledge, practices and abilities to one gender only. Gendered divisions, including cultural constructs of masculinity and generational dynamics, inform each of the identified sub-themes related to the role men played and could potentially play in newborn care. Men were not involved in physically carrying out care tasks however men did involve themselves in other ways and some showed a desire to increase their involvement.

### Perceptions of male involvement

#### Areas inaccessible to men

Certain areas – physical spaces or spheres of influence – were closed to men and male influence entirely with men playing a very limited or no role in performing care tasks. Men designated the delivery room and the immediate care of the baby after birth as “women’s affair” (*Male, 50)*, a space completely under female influence and control. Men were usually sent from the delivery room by attending women during the most active parts of labour and not called back to see mother and child until immediate newborn care tasks were completed:

He knows nothing about that [cleanliness of delivery room] because men are not supposed to be present when their wife is delivering. ... he said the women there would even ask you to go out and wait *(Male, 42).*

When his wife is giving birth he doesn’t enter the delivery room so he doesn’t know what happens to the baby and it has never occurred to him to think about it *(Male, 37)*.

Only two participants recounted men’s involvement in labour and delivery and only in unexpected or quickly progressing births. One participant repeatedly resisted her husband’s attempts to fetch a traditional birth attendant. In these instances men were present for labour and followed some instructions to fetch water, for example, but women still described delivering on their own without physically involving their husbands:

Although the husband was there when she was delivering…he could not help her (*Female, unknown)*.

Finally she delivered on her own at 7 am. Her husband went to stand outside when she started pushing. He went to the room when he heard the child crying *(Female, 33*).

Gendered divisions of labour place both genders in particular physical spaces: men at work, women at home. Although some women farm or trade they are still responsible for completing household duties and the household is perceived as their main domain. Women are seen as “naturally” closer to children, both physically and as a result of their gender:

Decisions concerning how newborns are cared for are mostly taken by women since they are closer to the babies than men (*Male, 37).*

He wouldn’t like to sit in such [newborn care home visits] because he sees that as ‘Woman/child and the worker’s affair’ (*Male, 28).*

Both men and women believe that men are less able than women to assume a caretaking role and women know more about birth and childrearing by nature of their gender. Interviews revealed the dismissive attitudes of some women towards a man’s ability to be involved in newborn care:

[Immediate newborn care] is done by the women who assist with the delivery. ‘They do everything and if the man says something they would ignore it and laugh at you (*Male,39).*

Men do not know anything about labour that is why we consult our mothers or anybody who has experience in childcare. If you rely on the men, you would die! (*Female, age unknown*).

#### No physical help in performing care tasks

In areas that were not entirely inaccessible to men, men were still not directly involved in performing newborn care tasks. Many men reported little to no physical contact with the newborn. Women and their mothers were responsible for physically carrying out tasks such as breastfeeding, cord care, bathing and keeping the baby warm. The potential for male involvement in encouraging or understanding breastfeeding was seen as inherently limited by biology:

He laughed [at the suggestion that he assist with breastfeeding]…He said men do not have breasts so the women decide when to breastfeed…‘I don’t get myself involved in these things’ (*Male, 39*).

For other care tasks gendered divisions of labour combined with some men’s perceived lack of knowledge resulted in little physical involvement of men with their newborn babies:

When the baby is very fresh [new], ‘I don’t hold it, because I wouldn’t know how to do that. So, I allow my wife alone to carry the baby’ (*Male, 50*).

The small number of men who did provide physical assistance participated in cord care under the instruction of a woman or carried the newborn while the mother completed other tasks:

He only assists with applying [methylated] ‘spirit’…on the cord if his wife asks him to (*Male, 39)*.

He assists his wife in bathing the babies by carrying one of them while the woman baths the other (*Male, 44, father of twins).*

#### Verbal instruction, support and supervision

Many men claimed involvement in newborn care through verbal instruction, support and/or supervision but often prefaced their comments by stating a lack of knowledge surrounding newborns. Men gave general reminders to complete tasks such as traditional cord care, keeping the baby warm and breastfeeding rather than offering specific advice:

From the little advice I know about child care, I tell her to practice them. For example if he realises that the baby is not covered well or is not wearing a sweater, he tells the wife to do it well or put a sweater on him from catching cold *(Male, 20).*

Only one male participant encouraged his wife to specifically adopt exclusive breastfeeding:

He learnt that babies should be fed exclusively that is why he insisted that his wife should practice that *(Male, 28)*.

Whereas men’s roles in most care practices were less active or ambiguous, it was common for men to bear a large responsibility in the care seeking and remedy of *asram -* a traditionally diagnosed and treated newborn illness widely believed not to respond to hospital treatment [29]. Men consistently sought out *asram* medicine from traditional healers and sometimes supervised the woman’s administration of the medicine:

The man plays a major role and makes sure that wherever that medicine to cure the child is could be found to help the child survive (*Male, 39).*

If the man brings the [asram] medicine he has to see to it that the woman uses it to treat the child. ‘Some women are lazy so if you don’t supervise them, they would not do the right thing’ (*Male, 20*).

Men’s active role in *asram* could be related to the need to purchase medicine which falls into their traditional role of provider described below.

Men also perform a supportive role by accompanying their wife and child in seeking care during or after delivery or running errands during delivery to purchase supplies at the instruction of traditional birth attendants or women’s mothers:

He’d also accompany his wife to send an *asram* baby to the hospital. Likewise, he’ll accompany his wife and a sick baby to the hospital to ensure that they get a better treatment *(Male, 44).*

The cord was cut with a new razor blade her mother in law sent her husband to buy when labour progressed *(Female, 20).*

#### Provider and decision maker

The most prominent ways in which men were involved in newborn care stem from their role as head of household defined by participants as a “duty” (*FGD1*) and “traditional responsibility” (*FGD2)* to ensure the welfare of mothers and children. Involvement as a husband/father was in some cases perceived as an obligation with moralistic undertones:

As a man, your wife and children’s welfare should be very important to you…Making sure proper treatment is given to your sick wife or newborn shows how responsible you are (*Male, 37).*

Two sub-themes emerged that define the traditional responsibilities of men: *decision maker* and *provider*. These intertwined roles are heavily facilitated by money in a mutually reinforcing relationship. Men are expected to provide money for the needs of women and children and at the same time, because men control money as head of household, they also assume the role of ultimate decision maker:

Every proper married woman should listen and do accordingly everything her husband tells her to do (*Male, 37).*

In reality this role of decision maker was often symbolic during delivery with women taking many of the decisions and having the power to order husbands to run errands or assist in seeking care at a facility in the case of complications:

If there were any complications she [the woman giving birth] would take the decision for her to be taken to hospital. The husband will go and look for a car and the cost would be borne by him (*Female*, *age unknown*).

Even where women make decisions men are ultimately held responsible for any “problems” and the potential to be blamed or incur monetary cost was a motivator for some men to provide verbal instruction, support and supervision:

Regular antenatal attendance is better than waiting until something happens only for husbands to be blamed or harshly spoken to *(FGD1)*.

He makes sure that the babies are breastfed well when he hears them crying and well-dressed to keep them from catching cold, because ‘If I don’t and they fall sick it’s going to be another wahala [trouble] for me’ (*Male, 44*).

Decisions that usually fell to men were around care seeking for illness, as this bore a monetary cost, and around *asram*, as this required leaving the house to seek medicine:

Men are the sole decision makers on [when a sick woman or newborn goes to the clinic] because the man has to look for money to send the woman and the baby to the hospital if they are sick (*Male, 20).*

The decision to go to the hospital would have been taken by the husband as he would have bared [sic] the cost (*Female, age unknown).*

As providers men were expected to attain money for the needs of women and children. A man’s authority as head of household was preserved and reinforced through his ability to provide for his family:

When a man is unable to meet his wife’s request due to financial constraints, the latter tends to ignore what he tells her (*FGD 1*).

While a few women used their own savings for some or all of the purchases related to newborn care supplies, the majority of women and all men reported that men alone paid for delivery costs, newborn care supplies and care seeking:

It is the man’s responsibility to start looking for money for the woman immediately after the woman informs him of the pregnancy… *(Male, 39).*

They [men] said [when a sick woman or newborn goes to the clinic] it is the duty of the husband. They give the women money to go to the hospital. *(FGD2).*

### Potential for increased involvement

Some men already encourage or engage in positive behaviours in collaboration with their wives including exclusive breastfeeding, institutional birth, and joint decision making. For example, one participant and his wife insisted on exclusive breastfeeding even when faced with strong community resistance:

He decided with his wife how the baby should be cared for. He cited the exclusive breastfeeding instance again where he had a lot of resistance from the community members including his mother-in-law but he ignored them because he knew what he was doing *(Male, 28).*

Many men expressed a desire, some very enthusiastically, to play more of a role in their newborn’s care and health. Increasing involvement in specific care practices was not discussed by male participants but some did mention increasing their handling of newborns because they wanted to “assist” or “give their wife a rest”. A lack of knowledge was frequently cited as the reason for current non-involvement and some men were motivated to learn more about their newborns to enable them to be involved:

He’d like to be involved in visits [by community health workers] himself to learn more about how to handle babies when they’re very small (*Male, 50*).

He…would want to be taught more…so that he can be able to take much care of his newly born child in order for his wife to have enough time to rest (*Male, 45*).

Many men also cited work- and money-related reasons for their inability to be involved. These were associated with lack of time, a need to make money and provide for the family as head of household and that involvement itself may have monetary implications:

He would have liked to be involved but because of the nature of his work…he wouldn’t even be at home to decide with them [his wife and mother-in-law]. He has to go and look for money to care for his wife and children because if he stays at home and they fall sick he cannot get money to send them to the clinic (*Male, age unknown)*.

I would like to be more involved if only I have money to do that…if you get yourself involved and the woman asks you to provide money, you can’t say no (*Male, 39*).

He would not say or suggest anything [about giving birth at a facility] because if he does the woman would ask him for money *(Male, age unknown)*.

Spaces closed to male influence are often reinforced by females, most especially the women’s mothers, highlighting generational power dynamics and the strong influence of elder women which can make it difficult for men to involve themselves in newborn care even if they are motivated. Many women had their mothers stay with them or traveled to their mother’s homes sometime during pregnancy, birth, or the postpartum period. Men expressed frustration with the dominating influence of some mother-in-laws and were discouraged from involving themselves in decision making processes because their contributions were disregarded. Some couples could make decisions together only after the mother-in-law ‘handed over’ authority:

When the baby is very young the woman’s mother would bath him for the first month and later hand him over to the mother. That is when the man can decide with the woman when to bathe the child (*Male, 20).*

Sometimes we [husband and wife] discuss something together and when [the women] meet their mother they take a different decision and they would not even tell you that this is what they have planned. It is very annoying so we just look at them to do what they like (*Male, 39)*

Some male participants’ attitudes towards increasing involvement in newborn care were not conducive to forming equitable, male–female partnerships to negotiate power, authority, and decision making. Rather, these attitudes towards increased involvement threatened to reinforce gender hierarchies and suggest negative impacts on women and/or the possible use of force:

The wife always takes his advice. If she doesn’t take it he will divorce her *(Male, 27).*

‘She would take it [my advice]! Why wouldn’t she take it?’ exclaimed one of the older participants who stressed that as a husband he has authority over his wife, and thus could not imagine why the latter should not take his advice *(FGD1).*

While a section of participants will use sustained persuasion and education [to get their wives to adopt new practices] other said they will use threats if persuasion fails (*Males, FGD2).*

## Discussion

Evidence from other areas of maternal and child health suggest that it is worth exploring the impact of male involvement interventions on newborn health. Intervention design requires a thorough understanding of socio-cultural contexts including men’s and women’s perceptions of male involvement and potential barriers or consequences of increasing male involvement. Our findings on newborn care from central Ghana suggest that men currently have little involvement in physically performing newborn care tasks but are involved as providers of money and as the decision maker for care seeking when mother or baby are sick. Some men reported providing verbal instruction and supervision but this was almost exclusively through general reminders, not specific health behaviour advice.

Our analysis confirms findings from other studies demonstrating that many men are interested in increased involvement in maternal and child health and that men already engage in some positive behaviors [8, 30, 31]. We found that many men embrace the importance of newborn care and that some already go against deeply entrenched cultural norms to carry out healthy practices such as exclusive breastfeeding, institutional delivery and joint decision making with their partners. A willingness to be involved is a necessary first step in behaviour change and men’s already-existing positive behaviours and attitudes should be built upon as entry points for supporting and increasing involvement along the entire continuum of maternal and child health care [30].

Studies have found that men’s positive attitudes regarding male involvement do not always translate to behaviour change [7, 15, 25]. It is important that the barriers identified in this and other studies are addressed in future intervention design and implementation. Gender norms were the main determinants of male involvement identified in our analysis. Constructions of masculinity in patriarchal societies often limit the ways in which men are “allowed” to engage in pregnancy, birth and child rearing. In many societies social space is not afforded to men who want to engage more in care taking and those who do are often stigmatized or discouraged [31]. Our findings on gender norms are consistent with other studies that have explored male involvement in other areas of maternal and child health and other barriers such as work obligations and lack of knowledge have also been identified elsewhere [7, 15–22, 25].

Our data suggests that women, both new mothers and senior women such as mother-in-laws, can be complicit in maintaining strict gender divisions and generational power dynamics that prevent men from increasing their involvement. This illustrates conclusions from other studies indicating that in many traditional societies senior women such as mother-in-laws, grandmothers and aunts wield a great amount of influence over maternal and child care practices, especially those concerning newborns and young children [32]. Wives and senior women with influence over newborn care, therefore, should also be engaged in exercises in gender transformation so they better understand the benefits of male involvement and can support men in expanding their roles as fathers.

The main barriers men face in wanting to increase their involvement in maternal and child health identified in this and other studies are described below, illustrating that barriers are multi-faceted and can exist simultaneously at different socio-cultural levels (see the ‘Barriers Men Face to Increasing Involvement’ subsection).

### Socio-cultural barriers men face to increasing involvement in maternal and child health^a^

#### Social

- Gendered divisions of labour and space, especially during pregnancy/birth/childrearing
- Maternal and child health spaces ‘inaccesible’ to men due to gender and/or generational dynamics
- Pressure to embrace dominant socio-cultural definition of masculinity
- No support for ‘alternative masculinities’ – men are ridiculed for stepping outside gender ‘boundaries’

#### Economic

- Men must work to support families financially, often required to travel away from home to work
- Employer and/or national policies do not allow men time from work during pregnancy, childbirth, postpartum periods

#### Institutional

- Health and gender programmes often only target women, men remain uneducated on relevant health behaviours
- Health facilities are not welcoming to/prohibit men

We found newborn care to be an area where women garner significant power and autonomy in a society where men hold most of the power. Some of the male participants conveyed attitudes affirming male dominance, some even suggesting they would use “force” to convince women to adopt certain practices. Increasing men’s abilities to influence certain spaces, most especially those limited spaces within which women are empowered in patriarchal societies, could lead to negative consequences for women such as decreasing women’s decision making power or increasing men’s use of force against their partner. Any increase in male involvement should safeguard against shifts in power which could inadvertently result in the dis-empowerment of women.

There are many ways in which men could be involved in newborn care interventions that range from giving reminders and providing support to actually carrying out care tasks. For example, interventions can engage with current constructs of masculinity by framing antenatal care, birth preparedness/complication readiness and performing care tasks to assist mothers as part of men’s traditional responsibility as head of household [12, 33, 34]. Alternative masculinities that include increased involvement can also be encouraged through peer networks, positive-deviance male role models and influential community leaders such as political and religious leaders or senior women [12, 13, 35]. Traditional constructions of masculinity are shown to have negative consequences for men by encouraging risk taking behaviour and discouraging health care seeking and emotional expression [2, 36]. By supporting alternative masculinities interventions could positively affect men as individuals, couple and family relationships, newborn health outcomes and community dynamics as a whole.

There were some limitations to our study. Interviews were not transcribed verbatim which limited our ability to analyze nuances of participant responses. The formative research was not specifically designed to explore male involvement; while men were asked questions on their involvement in newborn care women were not asked about their opinions on male involvement which limited our ability to incorporate their perspectives into our analysis. In addition, our secondary analysis was limited to available data, questions differed between women and men and could not be modified as research progressed and FGDs only included men. Future research specifically formulated to explore male involvement in newborn care, including more perspectives of women, could produce more relevant results. Despite these limitations however our data do provide a useful insight into the potential for future interventions.

## Conclusions

Men’s roles in newborn care in this context were perceived as mostly limited to the provision of money and some decision making surrounding care seeking. While many men were interested in increasing their involvement in newborn care they encountered barriers including gendered and generational divisions of labour and space. Male involvement alone should not be an outcome measure as male involvement can include a variety of behaviours, some of which may have little effect on performance of healthy practices and may even be detrimental to women. Rather, interventions using a male involvement strategy to improve maternal health require a thorough understanding of socio-cultural contexts and any potential negative effects increased male involvement could have on women’s autonomy. Strategies to include men in newborn care and other maternal and child health interventions should be mutually empowering for both women and men.

### Endnote

^a^Compiled from findings of other authors [8, 12, 19, 21, 23, 30, 31, 37, 38] as well as this secondary analysis.

## Abbreviations

FGD: Focus group discussion.
